# Halogenation and Dehalogenation Potential of Microorganisms in Yangtze River Waters

**DOI:** 10.3390/microorganisms13092133

**Published:** 2025-09-12

**Authors:** Zhixuan Wang, Lin Hu, Li Wang, Rulong Liu

**Affiliations:** College of Oceanography and Ecological Science, Shanghai Ocean University, Shanghai 201306, China; wangzhixuan1001@163.com (Z.W.); hulin9921@163.com (L.H.); l-wang@shou.edu.cn (L.W.)

**Keywords:** Yangtze River, halogenate, dehalogenase, metabolic potential, halogenated organic compounds, metagenome, genome

## Abstract

The discharge of pollutants into rivers has been increasing with the rapid industrial development and extensive agricultural use of pesticides and herbicides. Halogenated organic compounds (HOCs) represent a significant class of environmental pollutants. It has been found that microorganisms have the ability not only to degrade HOCs but also to synthesize them. Little is known about the halogenation and dehalogenation potential of microorganisms in river waters. In this study, we investigated the halogenation and dehalogenation potentials of microorganisms in the Yangtze River, which originates from the Tibetan Plateau, flows through southwestern, central and eastern China, and finally joins the East China Sea. A systematic metagenomic and bioinformatics analysis identified and quantified genes encoding four dehalogenases and two halogenases, providing fundamental data for the halogen cycle in the Yangtze River water body. The study showed that the microbial community in the Yangtze water body was mainly associated with dehalogenation potential, and the relative abundance of dehalogenase genes was higher than that of halogenase genes. Among the microorganisms with halogenation and dehalogenation potentials, Pseudomonadota and Actinomycetota dominated. Some microorganisms possessed both halogenation and dehalogenation functions, suggesting a potential adaptive strategy to environmental fluctuations. The presence of diverse and complete dehalogenation metabolic pathways highlights the microbial potential for bioremediation. These microorganisms not only contribute to the degradation of halogenated organic matter but also play crucial roles in carbon, nitrogen, and sulfur cycling. This study provides essential data for understanding microbial halogenation and dehalogenation potential in the Yangtze River, offering insights into the microbial-driven biogeochemical cycling mechanisms in its waters.

## 1. Introduction

Halogenated organic compounds (HOCs), such as polychlorinated biphenyls (PCBs), have remarkable physical-chemical characteristics such as high stability, semivolatility and hydrophobicity [1]. These compounds serve as biocides, intermediates for chemical synthesis, solvents and degreasing agents, among other applications [2]. Classified as persistent organic pollutants (POPs), anthropogenic HOCs are widely distributed in various environments [3] and can persist for extended periods [4], accumulating in the lipid tissues of organisms [5]. They not only cause serious impacts on aquatic ecosystems, but also pose significant risks to human health due to their carcinogenic, teratogenic and mutagenic properties. Traditionally considered fully anthropogenic, HOCs are now recognized to also occur naturally, with over 8000 natural HOCs have been identified [6].This indicated that HOCs are naturally occurring organics in the environment.

Microorganisms play a key role in the biogeochemical cycling of HOCs. On the one hand, some microorganisms can synthesize natural HOCs [7,8]. Non-phototrophic marine bacteria such as *Erythrobacter* and *Pseudomonas* strains were found to produce chloromethane [9]. In soil, extracellular enzymes released by bacteria and fungi contribute significantly to organic chloromethane formation [10], highlighting their role in HOC cycling. On the other hand, many microorganisms have the ability to degrade HOCs. It was found that marine members of the *Deltaproteobacteria* can perform reductive dehalogenation [11], while deep-sea *Chloroflexi* harbor genes encoding haloacetate dehalogenase, haloalkane dehalogenase, and (S)-2-haloacid dehalogenase, enabling HOC degradation [12]. These microorganisms convert HOCs into less toxic or harmless compounds through diverse metabolic pathways.

The Yangtze River, the third longest watershed in the world, covers one fifth of China’s land area. Originating from the Tibetan Plateau, the Yangtze River meanders eastward, traversing 11 provinces before ultimately merging into the East China Sea. It serves as a water source for one-third of China’s population and plays a significant role in the nation’s culture and economy. However, rapid economic growth in the Yangtze River Delta region has led to increasing pollution from industrial and agricultural activities [13]. The river’s banks host dense populations and chemical industries, with pollution sources often located near drinking water intakes, creating serious ecological challenges. Among many pollutants, HOCs have attracted widespread attention due to their persistence, potential for long distance migration, and biological toxicity in various environments [14].

Pollutants from land-based sources are primarily transported to the ocean through river runoff and extensive atmospheric transport, a process that has significant implications for global biogeochemical cycling. Previous studies have detected a wide variety of halogenated contaminants in the Yangtze River basin, including Cl/Br-polycyclic aromatic hydrocarbons (PAHs), chlorinated compounds, and other pollutants, indicating that riverine inputs are a major source of contamination to the Yangtze River estuary and adjacent sea areas [14,15,16]. The presence of dechlorinating bacteria in Yangtze River sediments has been reported [17]. However, limited information is known about the microbial dehalogenation and halogenation potential, distribution, and metabolic pathways in the water bodies, which limits our understanding of the mechanisms of halogenated organic matter cycling in the Yangtze River ecosystem.

This study systematically analyzed the taxonomic composition and functional metabolism of microbial communities involved in halogenation and dehalogenation processes across the Yangtze River’s upper, middle, and lower reaches. Through integrated metagenomic sequencing and bioinformatics approaches, we identified key microbial taxa and metabolic pathways driving these biogeochemical transformations in the Yangtze River. The results reveal critical microbial mechanisms driving halogen cycling, advancing our understanding of HOC transformation in aquatic ecosystems. These findings establish a vital framework for understanding microbial contributions to HOC biodegradation and bioremediation strategies in large river systems.

## 2. Materials and Methods

### 2.1. Metagenomic Data Acquisition

Our analysis leveraged publicly accessible metagenomic datasets (NCBI BioProject: PRJNA873262) [18] with explicit authorization obtained from the original data producers. The datasets encompass both particle-associated (WP, >2 μm) and free-living (WF, 0.22–2 μm) microbial fractions. Six strategically selected sampling sites spanned the Yangtze River’s longitudinal gradient: upper (e.g., Qinghai Province, high-altitude headwaters), middle (e.g., Wuhan City, transitional zone), and lower (e.g., Shanghai City, tidal estuary) reaches. These locations capture a gradient of anthropogenic pressures, including urbanization intensity, industrial activity, and gross domestic product (GDP), while simultaneously representing an altitudinal gradient from 4000 m to near-sea level.

### 2.2. Metagenomic Data Analysis

#### 2.2.1. Assembly

The raw data obtained from NCBI were subjected to quality control using Trimmomatic (v.0.39) [19] with parameters specified as “CROP:145, HEADCROP:10, LEADING:20, TRAILING:20, SLIDINGWINDOW:4:25, MINLEN:50”. And the clean reads were assembled into contigs using metaSPAdes (v.3.15.5) (-m 700) [20]. Coding sequences in the metagenomic data were predicted using Prodigal (v.2.6.3) (-p meta) [21]. Based on the prediction results, the non-redundant gene set was obtained by using CD-HIT (v.4.8.1) [22] with default parameters.

#### 2.2.2. Functional Annotation, Halogenase and Dehalogenase Genes Identification and Calculation of Their Relative Abundance

Functional annotation was performed by using BlastKOALA [23] against the Kyoto Encyclopedia of Genes and Genomes (KEGG) database. The coding sequences were aligned to the UniProt [24] protein database using blastp [25] (parameter: -outfmt 5,-max-target-seqs 10, other defaults). Reductive Dehalogenase was identified based on the Reductive Dehalogenase Database (https://rdasedb.biozone.utoronto.ca/ (accessed on 5 August 2024)) [26]. The protein sequences of halogenase and dehalogenase with coverage ≥ 90%, identity ≥ 60% and E values < 10^−5^ were selected for downstream analysis. The clean reads from each metagenome were mapped back to non-redundant gene set using BWA (v.0.7.17) [27]. Samtools (v.1.9) [28] was used to extract the number of mapped reads and length of each gene sequences, which was used to calculate the relative abundance of the genes, expressed as GPM (genes per million).

#### 2.2.3. Genome Binning and Dereplication

Metagenome-assembled genome (MAGs) were conducted using MetaBAT (v.2.12.1) [29], CONCOCT (v.1.1.0) [30] and DAS-Tool (v.1.1.6) [31], and the quality of the MAGs was assessed using CheckM (v.1.1.6) [32], which retained only those with completeness ≥ 50% and contamination ≤ 5%. Redundant MAGs were dereplicated using dRep (v.2.3.2) [33] at 95% average nucleotide identity (ANI). As a result, a total number of 150 nonredundant MAGs were selected for downstream analysis.

#### 2.2.4. Identification, Relative Abundance Calculation and Metabolic Pathway of Halogenated and Dehalogenated MAGs

Coding sequences in MAGs were predicted using Prodigal (v.2.6.3) [21]. Functional annotation was performed using BlastKOALA (v.1.3.0) [23] against the KEGG database with default parameters. The annotation results were manually checked to screen for MAGs with the metabolic potential for biogeochemical processes of HOCs, i.e., the MAGs containing genes encoding halogenase or dehalogenase. The sequences of halogenase and dehalogenase genes were aligned with the UniProt [24] protein database using blastp [25], and only MAGs containing halogenase and dehalogenase gene protein sequences with ≥90% coverage and ≥60% identity were selected as potential halogenating and dehalogenating microorganisms. A maximum likelihood phylogenomic tree was constructed using FastTree2 (v.2.1.11) [34] for the 63 MAGs containing halogenase or dehalogenase genes, and two Acidobacteriota genomes (GCF_000022565.1, GCF_000178955.2) were selected as the root. The phylogenomic tree was visualized with iTOL. Taxonomic classification of the MAGs was determined using GTDB-Tk (v.2.4.0) [35]. The relative abundance of each halogenation or dehalogenation MAG was calculated by mapping MAG sequences to clean reads of each metagenome using coverM (v.0.6.1) [36]. The metabolic pathways of 63 MAGs with halogenation or dehalogenation potentials were constructed based on the annotation results from PROKKA [37] and BlastKOALA (v.1.3.0) [23].

#### 2.2.5. Statistical Analysis

Statistical comparisons of enzyme distributions were conducted using the Wilcoxon rank-sum test in R (v.4.3.1) [38] to evaluate: (1) intra-habitat variations in relative abundances of distinct halogenase/dehalogenase subtypes, and (2) inter-habitat differences (WF vs. WP) for individual enzyme homologs. Significance thresholds were set at *p* < 0.05. Visualizations were generated using the ggplot2 package (v.3.5.1).

## 3. Results

### 3.1. Relative Abundance of Genes for Dehalogenases and Halogenases in Yangtze River Waters

**Figure 1:**
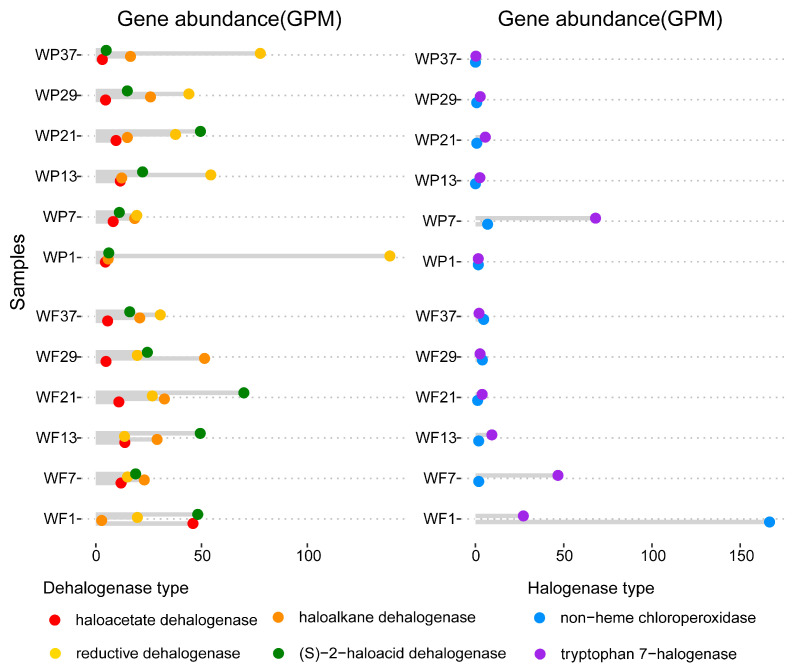
Relative abundance of dehalogenase and halogenase genes in Yangtze River water microbial communities (river direction from 1 to 37), comparing free-living (WF) and particle-associated (WP) fractions.

**Figure 2:**
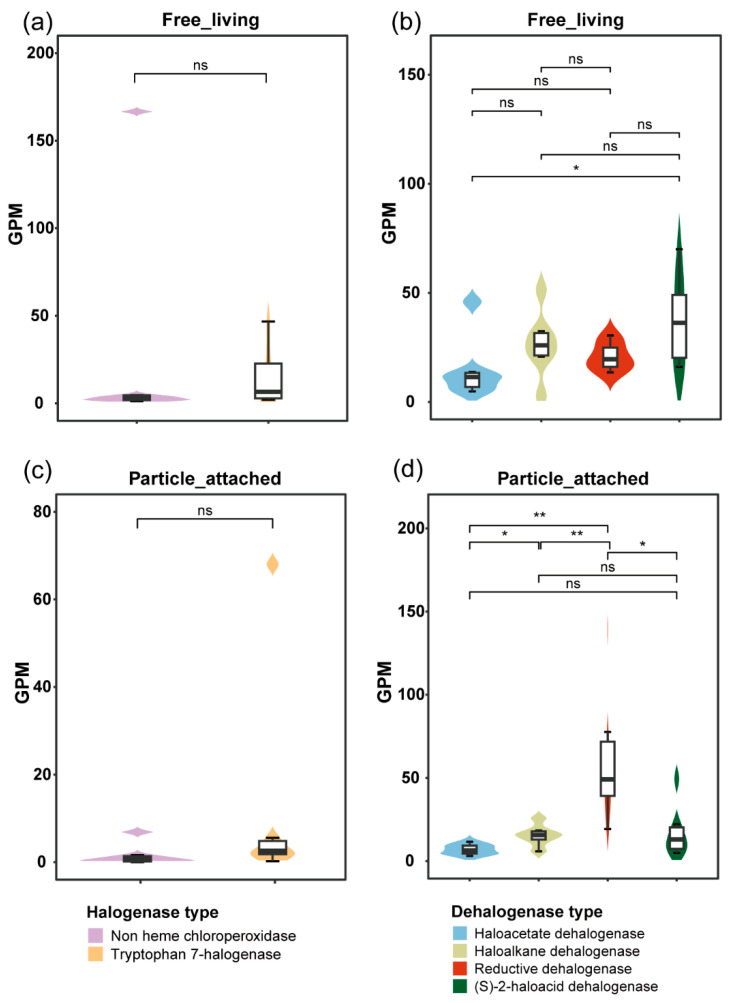
(**a**) Statistical analysis of the significant differences in the relative gene abundances (GPM) of non-heme chloroperoxidase and tryptophan 7-halogenase in WF. (**b**) Statistical analysis of the significant differences in the GPM of haloacetate dehalogenase, haloalkane dehalogenase, reductive dehalogenase, and (S)-2-haloacid dehalogenase in WF. (**c**) Statistical analysis of the significant differences in the GPM of non-heme chloroperoxidase and tryptophan 7-halogenase in WP. (**d**) Statistical analysis of the significant differences in the GPM of haloacetate dehalogenase, haloalkane dehalogenase, reductive dehalogenase, and (S)-2-haloacid dehalogenase in WP. Asterisks indicate statistically significant differences, *, *p* < 0.05, **, *p* < 0.01, and ns indicates no significant difference.

After the quality filtering, assembly, gene prediction and functional annotation, four types of dehalogenases and two types of halogenases were identified in the twelve sets of metagenomic data from the Yangtze River. The halogenases annotated in the samples of this study included non-heme chloroperoxidase (CPO) and tryptophan 7-halogenase (Figure 1). The relative abundance of tryptophan 7-halogenase genes tended to increase and then decrease in both WP and WF along the river direction (site 1 to 37) (Figure 1). Non-heme chloroperoxidase gene abundance displayed distinct trends: a decline followed by a slight increase in WF, and an increase followed by a decrease in WP. No significant differences in abundance were observed between WF and WP fractions for both halogenase (Figure 2).

**Figure 3:**
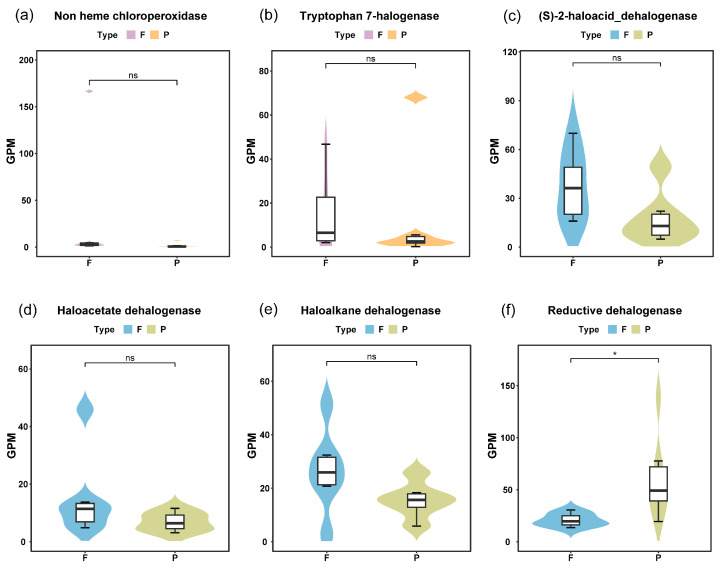
(**a**) Comparative analysis of the GPM of non-heme chloroperoxidase across distinct habitats. (**b**) Comparative analysis of the GPM of tryptophan 7-halogenase across distinct habitats. (**c**) Comparative analysis of the GPM of (S)-2-haloacid dehalogenase across distinct habitats. (**d**) Comparative analysis of the GPM of haloacetate dehalogenase across distinct habitats. (**e**) Comparative analysis of the GPM of haloalkane dehalogenase across distinct habitats. (**f**) Comparative analysis of the GPM of reductive dehalogenase across distinct habitats. Asterisks indicate statistically significant differences, *, *p* < 0.05, and ns indicates no significant difference.

The results also showed that dehalogenases, i.e., haloacetate dehalogenase, haloalkane dehalogenase, reductive dehalogenase, and (S)-2-haloacid dehalogenase, were prevalent in the 12 metagenomes (Figure 1). In the WP fraction, haloacetate dehalogenase abundance exhibited a unimodal distribution along the river continuum (sites 1–37), peaking at intermediate sites before declining, whereas WF showed a consistent downward trend (Figure 1). Haloalkane dehalogenase in WF exhibited an initial increase followed by decrease along the river course (1 to 37) (Figure 1). Comparative analysis between fractions revealed divergent patterns for reductive dehalogenase: WF maintained stable abundance levels along the river continuum (site 1 to 37), while WP showed a sharp decrease followed by gradual increase (Figure 1). Overall, the abundance of reductive dehalogenase was significantly higher in WP compared to WF (*p* < 0.05) (Figure 3f). (S)-2-haloacid dehalogenase abundance in WP displayed an initial increase-decrease pattern, whereas a decrease-increase-decrease fluctuation was observed in WF (site 1 to 37).

Significant inter-enzyme differentiation emerged within fractions. WF showed 3.4 folds higher gene abundance of (S)-2-haloacid dehalogenase than haloacetate dehalogenase (*p* < 0.05) (Figure 2). In contrast, WP exhibited significant differences (*p* < 0.05) between: (1) haloalkane dehalogenase and haloacetate dehalogenase (1:2.8 ratio), and (2) reductive dehalogenase and the other three dehalogenase types (6.4–12.7 fold higher) (Figure 2). Biofilm-like structures were formed on particulate organic matter [39], creating anaerobic microenvironments. These distinct niches were found to promote reductive dehalogenation (anaerobically associated), while microbial degradation processes released dissolved organic carbon (DOC), demonstrating significant impacts on Yangtze River carbon cycling.

### 3.2. MAG Reconstruction and Phylogenomic Analysis of MAGs with Halogenation and Dehalogenation Potential

**Figure 4:**
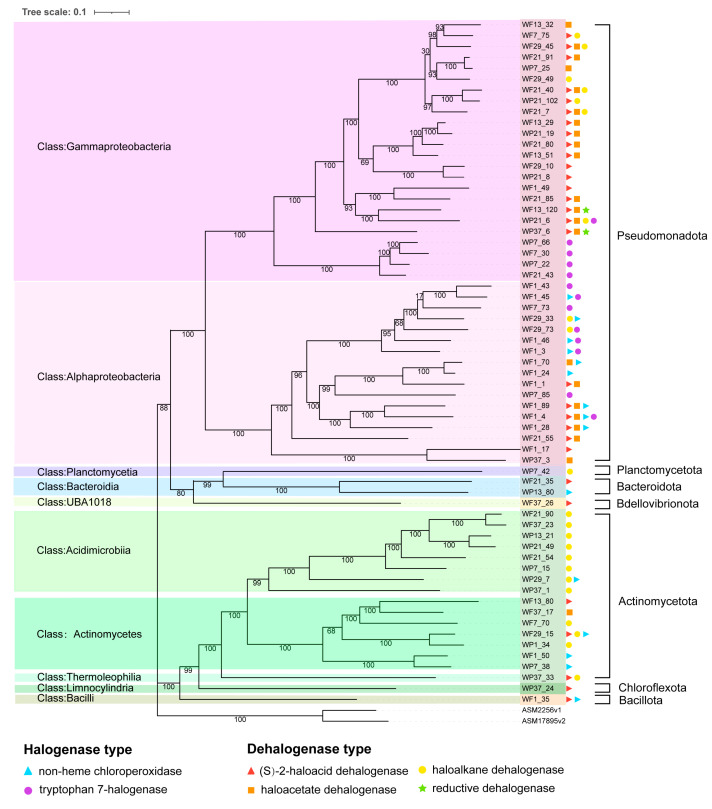
Maximum-likelihood phylogenomic tree of 63 MAGs encoding dehalogenase and halogenase genes, with functional annotations indicated by distinct marker symbols.

In this study, 2229 MAGs were reconstructed from the 12 metagenomes. Following dereplication at 95% average nucleotide identity (ANI), 150 non-redundant MAGs were obtained, among which 63 were identified as possessing potential for HOC cycling through halogenation and dehalogenation processes. Approximately two-thirds of these 63 genomes showed >80% completeness. A phylogenomic analysis of the 63 MAGs was conducted, revealing their classification into 7 phyla (Pseudomonadota, Planctomycetota, Bacteroidota, Bdellovibrionota, Actinomycetota, Chloroflexota, and Bacillota) and further assignment to 10 classes (Gammaproteobacteria, Alphaproteobacteria, Planctomycetia, Bacteroidia, UBA1018, Acidimicrobiia, Actinomycetes, Thermoleophilia, Limnocylindria, and Bacilli) (Figure 4).

Halogenase genes were identified in 26 MAGs, with 50% assigned to the class Alphaproteobacteria (phylum Pseudomonadota). Non-heme chloroperoxidase and tryptophan 7-halogenase genes were predominantly encoded by the orders Sphingomonadales, Enterobacterales, and Rhizobiales, both encoding oxidative halogenases (Figure 4, Table S1).

Dehalogenase genes were detected in 48 MAGs, with Pseudomonadota being the predominant phylum, represented by 20 Gammaproteobacteria and 10 Alphaproteobacteria genomes (Figure 4, Table S1). Actinomycetota constituted the second most abundant phylum harboring these genes (Figure 4, Table S1). (S)-2-haloacid dehalogenase and haloacetate dehalogenase were primarily distributed in Pseudomonadota, whereas haloalkane dehalogenase was predominantly associated with Actinomycetota (Figure 4, Table S1). Reductive dehalogenase genes were identified in only two MAGs, WF13_120 and WP37_6, both classified as Gammaproteobacteria (Pseudomonadota) (Figure 4, Table S1).

### 3.3. Relative Abundance of Halogenating and Dehalogenating Microorganisms

**Figure 5:**
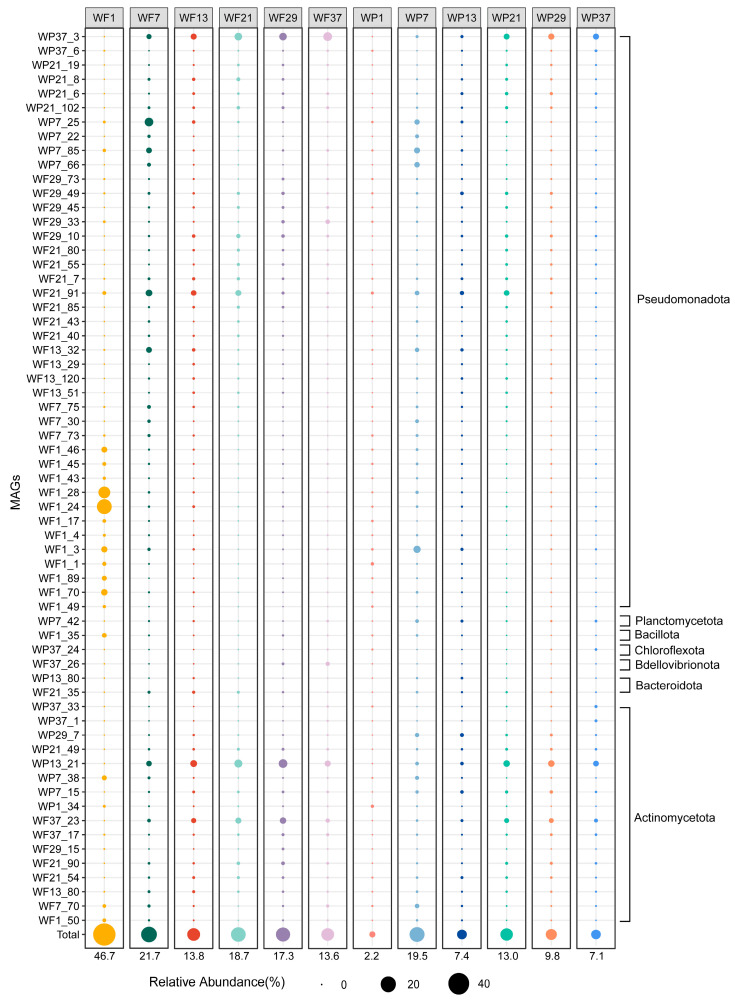
Relative abundance of dehalogenating and halogenating microorganisms in Yangtze River water communities, with dot size proportional to abundance values.

**Figure 6:**
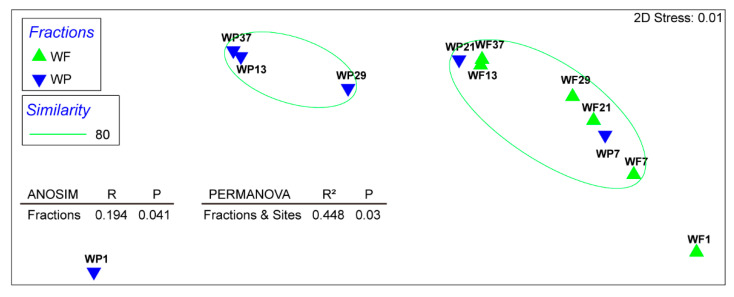
Non-parametric multi-dimensional scaling (nMDS) based on Bray–Curtis similarity matrix of relative abundances of microbial genomes involved in halogen cycling within each sample, with distinct symbols representing different habitats.

Relative abundance analysis of the 63 MAGs with halogenation/dehalogenation potential was performed across twelve Yangtze River metagenomes using CoverM, (v.0.6.1), revealing substantial variation (2–46.7% of total clean reads) of the population associated with HOC metabolisms (Figure 5). Non-metric multidimensional scaling (nMDS) revealed significant differences in microbial communities involved in halogen metabolism between WP and WF fractions (Figure 6). PERMANOVA further confirmed that community composition was significantly influenced by Sites and Fractions, together explaining 44.8% of the total variance (R^2^ = 0.448, *p* = 0.03). The overall relative abundance of the HOC related population was the highest in the WF1 sample (46.7% of total reads), followed by WF7, whereas WP1 exhibited the lowest abundance (Figure 5). Taxonomic profiling revealed Pseudomonadota as the predominant phylum in WF1, with WF7 maintaining secondary dominance. Within WF1, MAGs WF1_24 and WF1_28 of the class Alphaproteobacteria emerged as dominant populations. WF7 demonstrated notable abundance of a Gammaproteobacteria MAG (WP7_25). The highest relative abundance of Actinomycetota was observed in sample WF29, followed by WF21. The Acidimicrobiia member WP37_3 (p_Actinomycetota) demonstrated maximal relative abundance in sample WF29.

### 3.4. Metabolic Pathway of Halogenated and Dehalogenated Microorganisms

**Figure 7:**
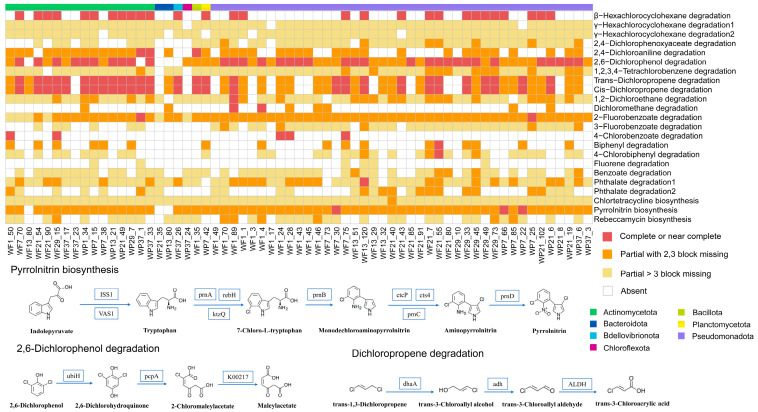
Pathway completeness analysis of dehalogenation and halogenation pathways in microbial MAGs. Categories: Complete/near-complete (≤1 enzyme missing), partial (≥2 enzymes missing), and absent (no enzymes detected).

Functional annotation of 63 genomes was conducted to assess metabolic pathway completeness in halogenating/dehalogenating MAGs (Figure 7). Metabolic pathways for nearly 20 compounds were identified, including chlorocyclohexane, chlorobenzene, chloroalkanes, chloroalkenes, fluorobenzoates, and polychlorobiphenyls (PCBs), demonstrating remarkable catabolic diversity. These findings highlight the pivotal role of Yangtze River microbial communities in HOC biogeochemical cycling through diverse degradation and synthesis capacities. The 29 MAGs harboring the β-hexachlorocyclohexane degradation pathway span four phyla and six classes, representing the major lineages observed in our dataset (detailed taxonomic information for all MAGs is provided in Table S1). Trans-1,3-dichloropropene degradation and Cis-1,3-dichloropropene degradation pathways were present in nearly half of the MAGs, with 10 containing complete pathways (harboring haloalkane dehalogenase, alcohol dehydrogenase, and aldehyde dehydrogenase). Five MAGs were annotated to the complete 2,6-Dichlorophenol degradation pathway, including 2-octaprenylphenol hydroxylase, 3-mercaptopropionate dioxygenase and maleylacetate reductase.

Biosynthetic pathways for antimicrobial compounds (including chlortetracycline, pyrrolnitrin, and rebeccamycin) were identified in these microbial communities, potentially conferring adaptive advantages in the heavily polluted Yangtze River ecosystem. Pyrrolnitrin biosynthesis was one of the halogenation pathways identified in our dataset. The nearly complete gene cluster for this pathway was found in three MAGs (WF7_30, WP7_66, and WP7_22). The highest pathway completeness was observed in WF1_89 and WF21_55, which belong to Alphaproteobacteria of Pseudomonadota. The biosynthetic pathways for chlortetracycline and rebeccamycin were partially annotated, though detection of core functional genes suggests these pathways may retain metabolic relevance.

**Figure 8:**
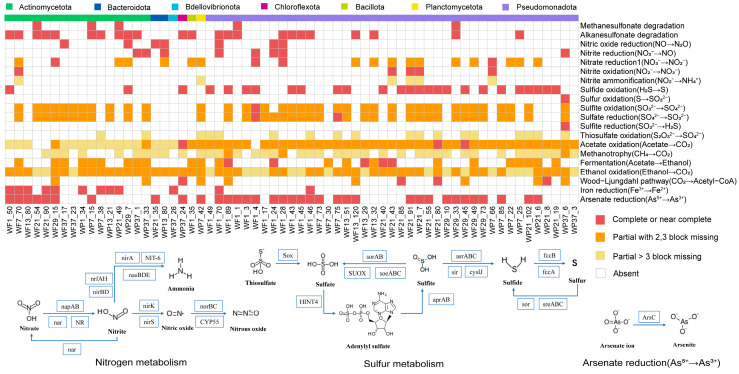
Metabolic pathway completeness analysis for carbon, nitrogen, sulfur, and other elemental cycles in microbial MAGs with dehalogenation/halogenation potential. Categories: complete/near-complete (≤1 missing enzyme), partial (≥2 missing enzymes), absent (no enzymes detected).

Additionally, we focused on the metabolic pathways related to carbon, nitrogen, sulfur, and other elements in the 63 MAGs (Figure 8). The arsenate reduction pathway (As^5+^→As^3+^) was identified in over half of the MAGs, with arsenate reductase annotated. Twenty-six MAGs possessed complete or near complete sulfide oxidation pathway (H_2_S→S), and the pathway involved in the sulfide dehydrogenase was annotated. Key enzymes involved in nitrogen oxide transformations-including nitric oxide reductase (NO→N_2_O), nitrite reductase (NO_2_^−^→NO), and ferredoxin-nitrate reductase (NO_3_^−^→NO_2_^−^)-were successfully annotated in these genomes.

## 4. Discussion

The Yangtze River microbial community exhibited a higher relative abundance of dehalogenase genes (e.g., (S)-2-haloacid dehalogenase) compared to halogenase genes (e.g., tryptophan 7-halogenase), as determined by the Wilcoxon rank-sum test (*p* < 0.01), suggesting that dehalogenation functions are more prevalent (Figure 1). This functional preference is consistent with microbial adaptation to halogenated organic compound (HOC) degradation, in accordance with established roles of dehalogenases in environmental detoxification under both oxic and anoxic conditions [40]. The highest (S)-2-haloacid dehalogenase abundance was observed in Wuhan (site 21), potentially reflecting local haloacid pollutant inputs from agricultural and pharmaceutical activities. The 2-haloacid dehalogenase is known to catalyze the conversion of 2-haloacids to less toxic 2-hydroxyacids [41], possibly mitigating environmental impacts from pesticide manufacturing byproducts. Notably, the Tibetan Plateau headwaters (site 1) exhibited the highest reductive dehalogenase gene abundance (Figure 1), likely due to anaerobic conditions favoring organohalide-respiring bacteria that employ reductive dehalogenases (*rdhAB*) for carbon-halogen bond cleavage [42]. This region also showed maximal relative abundance of the non-heme chloroperoxidase (CPO) gene, consistent with its role as a potential HOC reservoir where permafrost-derived organic matter enters aquatic systems [43]. Given CPO’s broad substrate spectrum [44], the halogenation processes it drives may be particularly important in this pristine ecosystem. Panzhihua, home to the largest steelmaking and titanium-vanadium production center in Southwest China, is considered as an important source of hydrocarbon input [45], which provides a source of carbon for the microorganisms in the Yangtze River, likely contributing to the relatively high abundance of halogenase genes at station 7, second only to site 1.

Through metagenomic assembly, 63 MAGs (metagenome-assembled genomes) with halogenation and dehalogenation potentials were obtained (Figure 4), covering 7 phyla and 10 orders, indicating a high diversity of microorganisms. All identified MAGs belonged to bacteria, suggesting that the halogen cycle in the Yangtze River may be mainly driven by bacteria. Pseudomonadota and Actinomycetota were identified as core functional taxa in the Yangtze River’ s halogen cycle. Pseudomonadota dominated the degradation of haloalkane (haloalkane dehalogenase) and haloacetate (haloacetate dehalogenase), while Actinomycetota prevailed in the metabolism of haloacids ((S)-2-haloacid dehalogenase), suggesting their critical roles in HOC removal (Figure 4). Beyond halogenated compounds, Pseudomonadota is implicated in diverse HOC degradation, including PAHs and polybrominated diphenyl ethers (PBDEs) [46], with demonstrated potential for chloroalkane/chloroalkene degradation [47]. Actinomycetota, renowned for secondary metabolite production [48], exhibit broad bioremediation capabilities, including petroleum hydrocarbon and pesticide degradation [49], likely induced by pollutant exposure [50].

The integrity and diversity of key dehalogenation metabolic pathways were found to represent valuable microbial resources for bioremediation applications. Complete degradation pathways for persistent pollutants, including β-hexachlorocyclohexane (β-HCH), were identified in several genomes (Figure 7). The associated haloalkane dehalogenases were shown to mediate cofactor-independent chlorine removal from β-HCH, significantly reducing its environmental toxicity [51]. The degradation of 2,6-dichlorophenol was demonstrated in almost genomes, suggesting its potential utilization as both carbon and energy sources by these microorganisms. Multiple dehalogenation pathways were identified in Yangtze River microbial communities, indicating broad substrate specificity for HOCs and previously unrecognized bioremediation potential. Halogenation metabolism was also observed, exemplified by pyrrolnitrin biosynthesis pathways. Bacterial sourced pyrrolnitrin has been shown as an important mechanism for biocontrol of fungal plant pathogens [52]. Biosynthetic pathways for both chlortetracycline and rebeccamycin were annotated. Genome-based functional annotation indicated the potential for biosynthesis of chlortetracycline, an antibiotic with significant applications in human therapeutics, livestock production, and aquaculture [53], and rebeccamycin, a clinically relevant antitumor agent [54]. These diverse metabolic pathways indicate that HOC synthesis and degradation can be mediated by microorganisms through multiple mechanisms, highlighting their potential for bioremediation applications and societal benefit.

Metabolic pathways associated with carbon, nitrogen, sulfur, and other elements were also examined across the microbial genomes. Arsenate reduction genes were mainly identified in MAGs of Burkholderiales (Figure 8), consistent with a previous report [55], indicating that arsenic (As) may influence microbial community composition. Sulfur metabolism, including sulfide oxidation [56], was detected in 26 MAGs, representing key processes for mineral transformation [57]. These findings highlight the metabolic versatility of Yangtze River microbiota, which, in addition to participating in halogen cycling, may also contribute to broader biogeochemical processes.

The coexistence of halogenation and dehalogenation functions observed in several MAGs (e.g., WP21_6 of Burkholderiales) may represent a potential adaptive strategy in dynamic environments. This hypothesis is consistent with previous metagenomic observations of co-occurring halogenase and dehalogenase genes [58]. Horizontal gene transfer may also be an important source of these halogenation and dehalogenation functions [59,60]. Ecologically, halogenation pathways may provide competitive advantages under nutrient-limited or competitive conditions through the biosynthesis of bioactive halogenated metabolites [58], whereas dehalogenation functions enable detoxification or utilization of halogenated compounds originating from both natural and anthropogenic sources [61]. The coexistence of these functions may therefore enhance microbial resilience in riverine ecosystems characterized by fluctuating pollutant inputs and redox conditions. While these findings underscore the ecological importance of bidirectional halogen cycling, the regulatory mechanisms governing this functional coexistence remain poorly understood. Future studies could focus on the regulatory mechanism of the co-expression of halogenase and dehalogenase genes, experimental validation of environmental conditions that promote dual functionality, screening microorganisms with dual functions of halogenation and dehalogenation, and developing novel microbial remediation technologies for the management of halogenated organic pollutants.

This study has several limitations that should be acknowledged. First, while we incorporated medium-quality MAGs (one third of MAGs, completeness 50–80%) to broaden genomic representation, their inclusion may have led to an underestimation of metabolic potential due to incomplete genome recovery. Additionally, metagenomic data alone cannot confirm in situ gene activity, and functional validation (e.g., metatranscriptomics or enzyme assays) would be required to assess actual microbial processes. Lastly, although the six sites were strategically selected to capture key ecological and anthropogenic influences, the number of sites is small compared to the enormous length of the Yangtze River and the temporal variations were not investigated. This means that the study provides a good pilot overview but cannot capture micro-scale heterogeneity or seasonal variation. Future work with expanded sampling, higher-quality genomes, functional validation, and co-analyzed environmental parameters (e.g., pollutant concentrations and other geophysical-chemical parameters) is needed to better resolve microbial drivers and activities.

## 5. Conclusions

In this study, the distribution characteristics and ecological significance of halogenation and dehalogenation functions among microorganisms in the water bodies of the Yangtze River were revealed by metagenomic analysis. It was found that the microbial community in the water body of the Yangtze River was characterized by dehalogenation function, with the relative abundance of dehalogenase genes higher than that of halogenase genes, indicating that the microorganisms were more inclined to degrade HOCs. Pseudomonadota and Actinomycetota were identified as the core functional taxa driving the halogen cycle in the Yangtze River. Notably, both halogenation and dehalogenation capabilities were identified within individual microbial taxa, which we propose as a hypothesis for a potential adaptive strategy to environmental fluctuations. However, this interpretation remains speculative and will require functional validation (e.g., transcriptomic or expression-based approaches) in future studies. Complete and diverse dehalogenation pathways were characterized, highlighting their bioremediation potential. Overall, these findings provide critical insights into microbial-mediated halogen cycling and offer a genomic baseline for future ecological and restoration-oriented studies.

## Data Availability

The assembled sequences of metagenome-assembled genomes (MAGs) are accessible in NODE (https://www.biosino.org/node (accessed on 11 September 2025)) with the accession number OEZ00021279 or through the URL: https://www.biosino.org/node/analysis/detail/OEZ00021279 (accessed on 11 September 2025).

## Supplementary Materials

The following supporting information can be downloaded at: https://www.mdpi.com/article/10.3390/microorganisms13092133/s1, Table S1: Genome quality and taxonomy of the 63 MAGs containing halogenate or dehalogenase.
